# Inhibitory Circuits in Cortical Layer 5

**DOI:** 10.3389/fncir.2016.00035

**Published:** 2016-05-06

**Authors:** Alexander Naka, Hillel Adesnik

**Affiliations:** ^1^The Helen Wills Neuroscience Institute, University of California BerkeleyBerkeley, CA, USA; ^2^Department of Molecular and Cell Biology, University of California BerkeleyBerkeley, CA, USA

**Keywords:** neocortex, barrel cortex, sensory cortex, layer 5, inhibition, interneuron, inhibitory microcircuits

## Abstract

Inhibitory neurons play a fundamental role in cortical computation and behavior. Recent technological advances, such as two photon imaging, targeted *in vivo* recording, and molecular profiling, have improved our understanding of the function and diversity of cortical interneurons, but for technical reasons most work has been directed towards inhibitory neurons in the superficial cortical layers. Here we review current knowledge specifically on layer 5 (L5) inhibitory microcircuits, which play a critical role in controlling cortical output. We focus on recent work from the well-studied rodent barrel cortex, but also draw on evidence from studies in primary visual cortex and other cortical areas. The diversity of both deep inhibitory neurons and their pyramidal cell targets make this a challenging but essential area of study in cortical computation and sensory processing.

## The Diversity of Layer 5 Excitatory Neurons

Before surveying the existent literature on layer 5 (L5) inhibitory neurons, we briefly review current knowledge on the connectivity and physiological properties of L5 pyramidal cells (PCs), as it provides important context for understanding L5 inhibitory circuits. One feature that distinguishes L5 from other cortical layers is the diversity of its PCs, which send a myriad of long-range projections to other cortical and sub-cortical structures (Lévesque et al., 1996; Veinante et al., 2000; Hattox and Nelson, 2007; Aronoff et al., 2010; reviewed in Harris and Shepherd, 2015). This simple anatomical fact establishes L5 as a primary cortical layer involved in the top-down control of other brain areas. Exactly how L5 circuits parse information to influence downstream circuits and control behavior is one of the central questions in neuroscience. Even though L5 is conventionally thought of as primarily an output layer, L5 PCs also receive direct thalamocortical input (Agmon and Connors, 1992; Meyer et al., 2010; Wimmer et al., 2010; Oberlaender et al., 2012; Rah et al., 2013) and can be driven by thalamic activity alone (Constantinople and Bruno, 2013), suggesting that L5 is an important input layer as well. L5 PCs also receive input from all cortical layers and are thus uniquely positioned to integrate nearly every local and afferent pathway in the cortex (Markram et al., 2015). Without exception, signals transmitted via these pathways invoke a mixture of synaptic excitation and inhibition (reviewed in Isaacson and Scanziani, 2011). Thus inhibition onto L5 PCs, the focus of this review, is crucial for nearly every aspect of L5 function.

**Figure 1:**
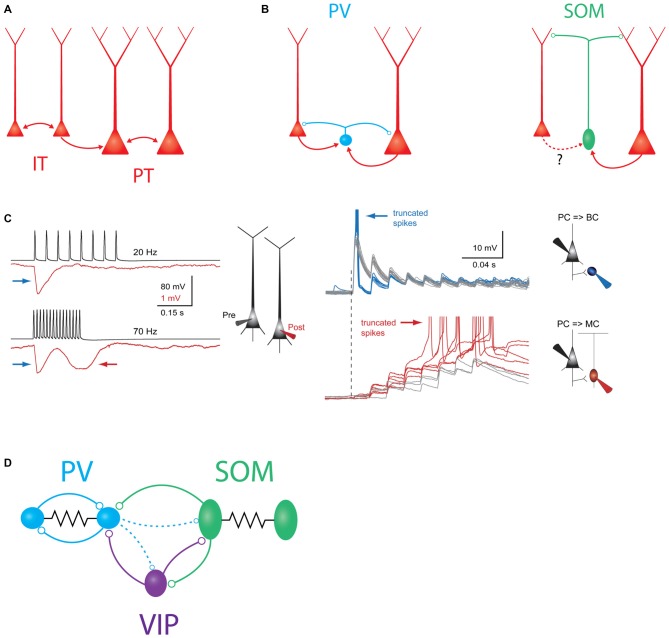
**Schematic overview of major intralaminar circuits in layer 5 (L5). (A)** Both the slender-tufted intratelencephalic (IT) cells (left) and thick-tufted pyramidal tract (PT) cells (right) form homotypic excitatory synaptic connections. IT cells additionally connect to PT cells, but PT cells connect to IT cells only very rarely. IT→IT and IT→PT connections both occur at a fairly high rate (Brown and Hestrin, 2009; Lefort et al., 2009; Kiritani et al., 2012). PT→PT connectivity occurs less frequently, but is structured into strongly interconnected subnetworks (Song et al., 2005; Perin et al., 2011). **(B)** Left: both IT and PT cells (red) excite parvalbumin (PV) cells (blue) and receive perisomatic inhibition from PV cells (Angulo et al., 2003; Silberberg and Markram, 2007; Kruglikov and Rudy, 2008). Right: somatostatin (SOM)/Martinotti cells (green) inhibit the distal dendrites of both PT and IT cells. These interneurons receive excitatory input from PT cells, but it is unknown if IT cells also excite them. **(C)** Experimental evidence for disynaptic inhibitory circuits between L5 pyramidal cells (PCs). Left: example traces showing two types of disynaptic inhibitory responses in a postsynaptic PC (red) driven by spiking in a presynaptic PC (black). Firing the presynaptic cell at 20 Hz (top traces) drives a transient, frequency-independent disynaptic inhibitory response (indicated by the blue arrow) which is likely mediated by activation of a PV/basket cell at the onset of spiking. Firing the same cell at 70 Hz (bottom traces) reveals a second, frequency-dependent form of disynaptic inhibition (indicated by the red arrow) which is likely due to delayed recruitment of a (SOM) Martinotti cell. Right: membrane potential responses of different interneurons to high-frequency stimulation of an L5 PC. Top: (PV) basket cells receive strong excitatory postsynaptic potentials (EPSPs) at the onset of stimulation, which can drive subthreshold (gray traces) or supra-threshold depolarization (blue traces). In either case, the postsynaptic response is initially strong, but then depresses rapidly. Bottom: EPSPs in Martinotti cells are weak and unreliable at the onset of L5 PC firing, but these facilitate and can eventually drive postsynaptic spiking, leading to frequency dependent disynaptic inhibition (FDDI; gray traces, subthreshold responses, red traces- suprathreshold responses). Reproduced with permission from Silberberg and Markram (2007). **(D)** Schematic of interneuron-to-interneuron connectivity in L5. PV cells (blue) form reciprocal chemical and electrical synapses with other PV cells. SOM cells are electrically but not chemically connected to other SOM cells, and form chemical synapses onto PV cells and vasoactive intestinal peptide (VIP) cells. VIP cells inhibit PV cells and SOM cells. Dashed lines indicate two weaker outputs from PV cells onto SOM cells and VIP cells.

L5 PCs can be broadly subdivided based on their projection targets into: (1) the pyramidal tract (PT) neurons, which project to subcortical regions, are located mainly in layer 5B, and display impressive “thick-tufted” apical dendritic morphologies; and (2) intratelencephalic (IT) neurons, which project mainly to other cortical regions and striatum, are found mainly in layer 5A, and have smaller and less complex dendrites (Hattox and Nelson, 2007; Larsen et al., 2008). PT and IT cells interconnect asymmetrically: while IT cells form excitatory synapses onto PT cells as well as other IT cells, PT cells preferentially connect to other PT cells (Brown and Hestrin, 2009; Lefort et al., 2009; Perin et al., 2011; Kiritani et al., 2012; Harris and Shepherd, 2015; Figure 1A). The two cell types are also distinguished by their intrinsic properties (Agmon and Connors, 1989, 1992; Chagnac-Amitai et al., 1990; Schubert et al., 2001), plasticity (Jacob et al., 2012; Greenhill et al., 2015), and more (Table 1). Furthermore, both PT and IT neurons can be further sub-divided based on their unique subsets of sub-cortical or cortical targets (Hattox and Nelson, 2007).

**Table 1:** **Properties and connectivity of layer 5 pyramidal neurons**.

Type (Other common nomenclatures)	Pyramidal Tract (PT) (Thick tufted, intrinsic bursting)	Intratelencephalic (IT) (Slender tufted/short, regular spiking)
**Morphology**
Larsen and Callaway (2006); Schubert et al. (2006); Oberlaender et al. (2011); Narayanan et al. (2015); and Ramaswamy and Markram (2015)	Broad, thick-tufted apical dendritic arbor, often with a prominent bifurcation in L4 or L2/3. Local axonal ramification is sparse and mostly restricted to infragranular layers, but spans multiple columns. Preferentially occupy L5B	Smaller dendritic arbor, with a slender apical tuft or no apical tuft. Local axonal ramification is dense, spans multiple columns and often ascends to supragranular layers. Preferentially occupy L5A
**Projection targets**
Wise and Jones (1977); Lévesque et al. (1996); Veinante et al. (2000); Hattox and Nelson (2007); and Groh et al. (2010)	Spinal cord, brainstem, superior colliculus, pontine nucleus, ipsilateral striatum, higher-order thalamus	Ipsilateral and contralateral striatum, contralateral S1, M1 and other cortical areas
**Intrinsic properties**
Larkum et al. (1999a,b, 2009); Schubert et al. (2006); Grewe et al. (2010); and Groh et al. (2010)	Can fire bursts or doublets, Prominent dendritic spiking, Non-adapting after burst	Regular or doublet spiking, Little dendritic excitability Adapting somatic spikes
**Local excitatory connectivity**
Markram (1997); Schubert et al. (2001); Schubert et al. (2006); Feldmeyer et al. (2005); Song et al. (2005); Krieger et al. (2007); Frick et al. (2008); Lefort et al. (2009); Hooks et al. (2011); Perin et al. (2011); Kiritani et al. (2012); and Kim et al. (2014)	Receive excitatory input from PCs in all cortical layers; strong inputs from L3, L5PT Engage in reciprocally connected subnetworks with other PT cells but rarely connect to L5 IT	Receive excitatory input from PCs in all cortical layers; strong inputs from L2, L4, L5IT, L6 corticothalamic cells Reciprocally connected to other L5 IT; provide excitation to L5 PT
**Long-range inputs**
Agmon and Connors (1992); Petreanu et al. (2007); Petreanu et al. (2009); Wimmer et al. (2010); Oberlaender et al. (2012); Xu et al. (2012); Constantinople and Bruno (2013); and Rah et al. (2013)	Strong input from ventral posteriomedial nucleus of thalamus (VPM) Weak input from posteromedial nucleus of thalamus (POm) Primary motor cortex and other cortical areas	Weak or no input from VPM Strong input from POM Primary motor cortex and other cortical areas

As a whole, L5 PCs have distinct physiological properties *in vivo* that distinguish them from excitatory neurons in other layers. First, L5 PCs display very broad sensory tuning (Brecht et al., 2003; Manns et al., 2004; de Kock et al., 2007; Sakata and Harris, 2009; Kim et al., 2015; Lur et al., 2016). Second, L5 PCs, especially PT cells, fire at high rates both spontaneously and during sensory responses (de Kock et al., 2007; Sakata and Harris, 2009; O’Connor et al., 2010; Hires et al., 2015). Third, many L5 PCs exhibit reductions in their firing rate during sensory stimulation or behavior, a feature not often seen in other cortical neurons, which might serve to expand their dynamic range (Krupa et al., 2004; Pluta et al., 2015; Sofroniew et al., 2015). These properties suggest that L5 PCs employ a dense coding strategy (reviewed in Harris and Mrsic-Flogel, 2013), which stands in contrast to the sparse code that has been observed in L2/3 (Brecht et al., 2003; Kerr et al., 2007; Crochet et al., 2011; Clancy et al., 2015; Peron et al., 2015) and other layers (de Kock et al., 2007; de Kock and Sakmann, 2009; O’Connor et al., 2010; reviewed in Barth and Poulet, 2012). L5 PCs are highly intrinsically excitable, and can integrate excitatory inputs from many different sources—both of which probably help establish this dense code (Schubert et al., 2001, 2006; Hooks et al., 2011; Zarrinpar and Callaway, 2016; Schnepel et al., 2015). Another major factor is the diverse cast of inhibitory circuits impinging onto L5 PCs.

## Major Subtypes of Layer 5 Inhibitory Neurons

Cortical interneurons can be broadly sub-divided into distinct cell types based on their morphology, connectivity, molecular and developmental identity, and electrophysiological and synaptic properties (Markram et al., 2004; Ascoli et al., 2008; DeFelipe et al., 2013). Increasingly, these cell types are being linked to specific functional specializations (reviewed in Gentet, 2012; Petersen and Crochet, 2013; Kepecs and Fishell, 2014; Petersen, 2014; Roux and Buzsáki, 2015; Womelsdorf et al., 2014). Cortical inhibitory interneurons can be grossly separated into three essentially non-overlapping groups based on their expression of the molecular markers parvalbumin (PV), somatostatin (SOM), or the serotonin receptor 5HT3aR (Rudy et al., 2011). Each molecular group can be subdivided into multiple cell types, though exactly how many remains unclear. The diversity of inhibitory cell types in the neocortex has been reviewed extensively elsewhere (Markram et al., 2004; Ascoli et al., 2008; Rudy et al., 2011; Huang, 2014; Kepecs and Fishell, 2014; Kubota, 2014; Taniguchi, 2014), so we will limit our discussion to the most relevant aspects for inhibitory circuits in L5 (Supplementary Table 1).

### PV Neurons

PV-expressing GABAergic neurons constitute the largest sub-class of L5 interneurons (Gonchar et al., 2008; Lee et al., 2010; Xu et al., 2010). All PV cells share a distinctive “fast-spiking” electrophysiological phenotype. In addition to their eponymous fast action potentials (sometimes also called thin or narrow), these neurons also display little or no spike-frequency adaptation, rapid membrane kinetics, and rapid synaptic conductances, which collectively allow them to fire precisely timed spikes at high rates (Hu et al., 2014). Importantly, the fast-spiking phenotype has allowed experimenters to distinguish PV cells from other types of neurons (such as PCs or SOM interneurons, which typically have broader spikes) during extracellular recording *in vivo*. The vast majority of information we have on L5 interneurons *in vivo* primarily derives from this type of analysis.

Most PV cells in L5 are basket cells whose axons densely target the perisomatic compartments of PCs, allowing them to impose rapid and powerful inhibition on the surrounding network (Xiang et al., 2002). Anatomically, basket cells take on a range of morphologies that have been described as “large”, “small” and “nest” derived from the unique and diverse structure of their intracortical axons (Gupta et al., 2000; Wang et al., 2002), and these different axonal phenotypes may correlate to functional distinctions (Buchanan et al., 2012; Bortone et al., 2014). However the differential circuit connectivity and function of these various basket subtypes remain to be fully elucidated.

Chandelier cells are the second major subtype of PV cells. These neurons have a highly distinctive and characteristic axonal morphology and primarily synapse on PC axons. Yet they are technically challenging to study due to their relative scarcity and current barriers to specific genetic access to them (Taniguchi et al., 2013; Huang, 2014). While chandelier cells are GABAergic, in some conditions they can actually depolarize postsynaptic PCs due to the locally elevated chloride reversal potential in the axon initial segment. However, the questions of whether and when chandelier cells exert excitatory or suppressive effects on their postsynaptic targets remain open (Szabadics et al., 2006; Glickfeld et al., 2009; Woodruff et al., 2010, 2011). Interestingly, while chandelier cells have been observed to occupy the infragranular layers (Inda et al., 2009; Taniguchi et al., 2013), to the best of our knowledge no direct evidence of connections from L5 chandelier cells onto L5 PCs has been published; these may be comparatively rare (Peters et al., 1982). However, connections onto L5 PCs have been observed originating from chandelier cells located in the supragranular layers, where they are more common (Jiang et al., 2013; Lee et al., 2015).

### SOM Neurons

The second largest class of L5 GABAergic neurons are SOM cells. In contrast to PV neurons, SOM cells primarily target the dendrites of excitatory neurons and receive facilitating excitatory input (Reyes et al., 1998; Markram et al., 2004; Silberberg and Markram, 2007). The best-studied type of SOM cell is the Martinotti cell, which is a subclass found across layers 2–6, but especially prevalent in L5. The axons of L5 Martinotti cells characteristically ascend to upper cortical layers, particularly to L1, where they ramify and form a dense axonal plexus innervating the distal apical dendrites of L5 PCs. Some Martinotti axons also target L4, and overall Martinotti cells display considerable morphological and molecular heterogeneity (Wang et al., 2004; McGarry et al., 2010). This heterogeneity has been demonstrated in a study comparing two transgenic reporter mouse lines (the “X98” line and the more commonly used “GIN” line), which appear to label distinct sets of Martinotti cells with mostly non-overlapping phenotypes (Ma et al., 2006). Another reporter, the “X94” line, labels a third subset of non-Martinotti SOM cells present in both L5 and L4 which display a quasi-fast-spiking electrophysiological phenotype and target their axons almost exclusively to L4 rather than L1. A distinctive feature of X94 cells in L5 is that they receive robust excitatory input from thalamocortical axons, indicating that they may play a key role in feed-forward inhibition from thalamus to cortex (Porter et al., 2001; Tan et al., 2008). On the other hand, Martinotti cells receive little or no thalamocortical input in the mature animal (Cruikshank et al., 2010; Ji et al., 2015), though recent results indicate that they may play a transient role in feedforward inhibition during development (Marques-Smith et al., 2016; Tuncdemir et al., 2016).

### 5HT3aR Neurons

The last major group of L5 GABAergic neurons express the serotonin receptor, 5HT3aR (Lee et al., 2010; Vucurovic et al., 2010). Even more so than L5 PV and SOM neurons, this sub-group is highly heterogeneous. While 5HT3aR neurons represent half of the GABAergic cells in L2/3, they comprise only a small fraction (~10–25%) of L5 interneurons (Lee et al., 2010; Xu et al., 2010; Rudy et al., 2011). This group includes interneurons expressing the molecular marker vasoactive intestinal peptide (VIP). These neurons have bipolar or bitufted dendritic morphologies and vertically oriented axonal arborizations (Vucurovic et al., 2010; Prönneke et al., 2015). In L2/3, VIP cells have recently been shown to target other interneurons as a part of dedicated disinhibitory circuits. However, VIP cells in L5 appear morphologically distinct from those in upper layers and comparatively little is known about them (Prönneke et al., 2015). Other 5HT3aR expressing interneurons, such as neurogliaform cells, are known to exist but rarely observed in L5 (Oláh et al., 2009; Jiang et al., 2015). Overall, data on 5HT3aR interneurons in L5 are scarce and much further experimentation is needed.

## Recurrent, Intralaminar Inhibition Within Layer 5

### Somatic Inhibition

PV basket-type interneurons mediate a major component of recurrent inhibition within L5. L5 PCs powerfully excite L5 PV cells (Angulo et al., 2003; Jin et al., 2014; Jiang et al., 2015; Pluta et al., 2015) and likely do so in a highly convergent and non-selective manner (Bock et al., 2011; Hofer et al., 2011; Scholl et al., 2015; all in L2/3 of V1). In turn, L5 PV cells connect onto surrounding L5 PCs at a very high rate, and diverge massively, with one recent study estimating that each L5 PV cell inhibits >1000 L5 PCs (Packer and Yuste, 2011, Figure 1B). This has led to the proposal that PV cells may provide a blanket of dense, non-specific inhibition to all excitatory cells (Fino et al., 2013). Despite this promiscuous connectivity, some studies suggest that the PV population may preferentially inhibit specific PC subtypes (Ye et al., 2015), though reports in L5 conflict over whether IT or PT cells receive more inhibition (Fariñas and DeFelipe, 1991; Lee et al., 2014; Rock and Apicella, 2015). Unlike Martinotti cells (see below), PV cells generally require multiple, co-occurring excitatory inputs in order to spike. However, they are more numerous than Martinotti cells in L5, and paired recordings suggest that the conductance of a PV to PC synapse in L5 is several fold larger than that of a Martinotti to PC synapse (Xiang et al., 2002; Kruglikov and Rudy, 2008), though this is likely partially confounded by the limited ability of somatic recordings to measure distal conductances (Williams and Mitchell, 2008). Thus, PV basket cells are poised to exert direct control over the spiking output of PCs and are likely the dominant inhibitory force in L5.

PV to PC inhibition is likely to be preferentially important during certain moments of cortical activation by sensory stimuli. Excitatory synapses onto PV cells are powerful, but exhibit prominent synaptic depression, and the outputs of PV cells onto L5 PCs depress substantially during trains of action potentials (Galarreta and Hestrin, 1998; Xiang et al., 2002; Silberberg and Markram, 2007; Figure 1C). Thus, as has been demonstrated in the hippocampus and other cortical circuits, PV neurons are typically recruited extremely reliably upon the first action potential in a PC spike train, but may stop firing shortly thereafter (Pouille and Scanziani, 2001; Gabernet et al., 2005). Due to these temporal dynamics, PV-mediated somatic inhibition may impose a temporal window on synaptic integration in L5 PCs, preventing summation of non-coincident inputs. The net consequence of this would be to precisely time the first few spikes in a sensory-driven PC spike train (Silberberg and Markram, 2007), similar to what has been shown for PV cells in L4 for thalamocortical input (Gabernet et al., 2005). The enforcement of such a precisely timed integration window may contribute to neuronal tuning in the whisker system (Wilent and Contreras, 2005), and enable temporal coding of stimulus features such as texture or object location (Petersen et al., 2002; Jadhav et al., 2009; Pitas et al., 2016). In agreement with this notion, *in vivo* recordings show that fast-spiking (putatively PV) cells in L5, and especially L5B, encode a high degree of information about the temporal features of a sensory stimulus and may play an important role in initiating precisely timed sequences of spikes during sensory responses (Reyes-Puerta et al., 2015a,b).

### Dendritic Inhibition

While PV neurons chiefly inhibit the soma and proximal dendrites of L5 PCs, SOM cells are thought to primarily target PC dendrites. In fact, one of the best understood recurrent inhibitory circuits within L5 is a motif in which L5 Martinotti cells mediate frequency dependent disynaptic inhibition (FDDI) between L5 PCs. FDDI is generated when an L5 PC fires a high-frequency burst of action potentials and excites a postsynaptic L5 Martinotti cell (Silberberg and Markram, 2007, Figure 1C). Because excitatory synapses onto Martinotti cells undergo strong short term facilitation, high frequency input from only one or a few PCs is sufficient to drive spiking in the Martinotti cell and inhibit the dendrites of nearby PCs (Kapfer et al., 2007; Silberberg, 2008; Berger et al., 2010; Kwan and Dan, 2012). This motif is widespread and occurs in the visual, auditory, motor, and prefrontal cortices in addition to somatosensory cortex (Berger et al., 2009).

The FDDI motif allows a small number of PCs to spread inhibition widely to the surrounding network—as few as four L5 PCs firing at high frequency is enough to drive inhibition in virtually all nearby L5 PCs (Berger et al., 2010). FDDI seems to recruit a relatively small number of L5 Martinotti cells, but these diverge extensively onto the surrounding PC network (Fino and Yuste, 2011; Jiang et al., 2015). FDDI-mediated connections between PT cells appear to be structured, since FDDI occurs reciprocally between two PT cells much more often than would be expected by chance (Berger et al., 2009). This enhanced reciprocity is reminiscent of motifs found in excitatory connectivity between PCs, and suggests that the indirect connectivity created by FDDI might complement the structured subnetworks that have been observed in cortical microcircuits (Yoshimura et al., 2005; Kampa et al., 2006; Perin et al., 2011). At first glance, this result is difficult to reconcile with the promiscuous, non-selective outputs of SOM cells (Fino and Yuste, 2011); this could potentially be resolved by specificity in excitatory connectivity onto SOM cells (Yoshimura and Callaway, 2005; Otsuka and Kawaguchi, 2009, 2013), or by “soft” structure in the synaptic weights of a densely connected PC-SOM network that shapes how Martinotti cells are recruited.

While FDDI can also occur outside of L5 (Kapfer et al., 2007), FDDI within L5 appears to be specific to PT (thick-tufted) PCs, since it is not observed between pairs of IT cells in L5 (Le Bé et al., 2007). The mechanism of this specificity is unclear, since Martinotti cells seem to target pyramidal cells non-selectively (Fino and Yuste, 2011; Jiang et al., 2015), and both PT and IT cells in prefrontal cortex receive equal amounts of SOM-mediated inhibition (Lee et al., 2014). One possibility is that L5 IT cells are simply less efficacious than PT cells at recruiting Martinotti cells to spike (Figure 1B). Future experiments utilizing paired recordings between identified L5 IT cells and Martinotti cells could resolve this. Another open question is whether PT cells can drive Martinotti-mediated FDDI onto the dendrites of L5 IT cells. If so, this disynaptic motif would represent an interesting inversion of the asymmetric connectivity from IT cells onto PT cells. Emerging evidence suggests that indirect circuits of this sort underlie stereotyped inhibitory interactions between excitatory cell types in cortex (Adesnik and Scanziani, 2010; Olsen et al., 2012; Xue et al., 2014; Yamawaki and Shepherd, 2015); these circuits might be thought of as inhibitory “pathways” in the cortical microcircuit (Naka, 2015) and will be an important topic for future study.

More broadly, FDDI might represent an avenue for PT cells to broadcast inhibition throughout the local microcircuit. While PT cells receive and integrate input widely, they do not locally excite many other excitatory neurons, except for other PT cells (Brown and Hestrin, 2009; Lefort et al., 2009; Harris and Shepherd, 2015; Jiang et al., 2015; Yamawaki and Shepherd, 2015). However, by harnessing the massive divergence of Martinotti cells, L5 PT cells can potentially route inhibition to a large cohort of neurons across multiple layers. Burst firing by one or a small number of L5 PT cells might therefore represent a “call to order”, quieting activity throughout an entire cortical column by activating FDDI.

FDDI may serve several functional roles during sensory processing. Most simply, it can act as negative feedback, inhibiting PCs in response to sustained epochs of high activity. More subtly, it can alter synaptic integration in L5 PCs by suppressing electrogenic events in the dendrites. The apical dendrites of L5 PCs can couple with the somatic compartment via dendritic calcium and NMDA spikes (Larkum et al., 1999b; Larkum, 2013; Major et al., 2013), which are highly sensitive to dendritic inhibition (Larkum et al., 1999b; Larkum and Zhu, 2002; Marlin and Carter, 2014). By suppressing the initiation of dendritic spikes FDDI could modulate the gain of the input/output function in L5 PCs (Larkum et al., 2004; Murayama et al., 2009), influence temporal correlations between L5 PCs (Berger et al., 2010), and shape tuning (Lavzin et al., 2012; Xu et al., 2012). In the same vein, since dendritic spikes are thought to be crucial for burst spiking, particularly in PT cells, FDDI can control the firing mode of L5 PCs by reducing burst firing. Lastly, since L5 PC apical tufts in the barrel cortex are major targets of “top-down” axons from higher cortical areas, including from frontal and motor areas (Petreanu et al., 2009, 2012; Mao et al., 2011; Manita et al., 2015), FDDI may act as a gate for feedback input to the primary somatosensory cortex. Since corticocortical feedback axons convey contextual and behavior related information to S1, FDDI is in a position to powerfully influence how context and brain state influence sensory processing (Larkum, 2013).

In addition to directly hyperpolarizing or shunting their postsynaptic targets via ionotropic GABA_a_ receptors, a recent study has shown that SOM cells in L2/3 can also reduce the probability of release at pyramidal-to-pyramidal synapses via a form of GABA_b_ mediated presynaptic inhibition (Urban-Ciecko et al., 2015). It is not yet known if this phenomenon affects excitatory transmission onto L5 PCs, nor if L5 SOM cells also engage in this form of inhibition.

### Inhibition onto Other Interneurons in L5

L5 interneurons also form circuits with other interneurons. L5 PV cells form both electrical and chemical synapses with other PV cells at a high rate (Galarreta and Hestrin, 1999, 2002). These densely coupled PV networks have been implicated in driving network synchrony and generating oscillations (Hestrin and Galarreta, 2005). In contrast, SOM cells generally seem to inhibit each other rarely or not at all, but do inhibit PV cells (Ma et al., 2012; Pfeffer et al., 2013; Zhang et al., 2014; Jiang et al., 2015; Tuncdemir et al., 2016). This asymmetric connectivity pattern among interneurons suggests that L5 SOM cell activity might redistribute inhibition along the somato-dendritic axis of L5 PCs, driving dendritic inhibition while simultaneously disinhibiting the somatic compartment by suppressing PV cells. SOM cells also inhibit other interneurons, including VIP cells (Pfeffer et al., 2013; Chen et al., 2015; Jiang et al., 2015).

In L2/3, VIP cells have emerged as specialists for disinhibition, which can release PC networks from inhibition by selectively suppressing other interneurons, especially SOM cells (Lee et al., 2013; Pfeffer et al., 2013; Pi et al., 2013; Fu et al., 2014; Karnani et al., 2016a,b). This effect has not yet been investigated in deeper layers, but seems likely to occur in some form. In L5, both PV and SOM cells are innervated by VIP inhibitory synapses (Dávid et al., 2007; Pfeffer et al., 2013). In the case of PV cells, VIP boutons preferentially target the soma, suggesting that VIP-mediated inhibition likely has a powerful influence over PV spiking in the same manner that PV neurons themselves yield control over PC spiking (Hioki et al., 2013). While VIP neurons are comparatively scarce in L5, the axons of L2/3 VIP neurons descend into deeper layers and connect to L5 PCs and interneurons (Jiang et al., 2013; Walker, 2016).

The set of interneuron-to-interneuron connectivity motifs described above has recently emerged as a “canonical” motif in cortical microcircuits (Figure 1D). However, other circuits outside of this scheme are also known to exist. Within L4 of the barrel cortex, PV cells provide dense reciprocal inhibition back onto SOM cells. Studies in V1 indicate that PV to SOM connectivity is much weaker in L5 (Pfeffer et al., 2013; Jiang et al., 2015), but it may still be fairly strong in L5 of the barrel cortex (Walker, 2016). Furthermore, PV cells also inhibit VIP cells to some extent in barrel cortex and V1 (Staiger et al., 1997, 2002; Pfeffer et al., 2013; Karnani et al., 2016b). Thus connectivity between these classes of interneurons is actually all-to-all, albeit with some connections being much stronger than others.

## Feed-Forward, Translaminar Inhibition of L5

**Figure 2:**
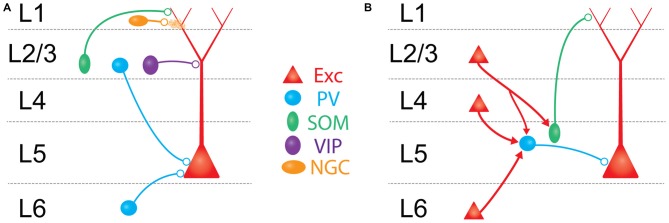
**Schematic overview of major translaminar inhibitory circuits impinging on L5 PCs. (A)** Inhibition arises from multiple types of interneurons outside of L5. In L1, 5HT3aR/Neurogliaform cells (NGCs, mustard) inhibit L5 PCs via volume release of GABA. In L2/3, SOM/Martinotti cells (green) and VIP cells (purple) target the apical dendrites of L5 PCs, while PV/basket cells (blue) synapse onto the perisomatic compartment via descending axons. In L6, translaminar PV/basket cells inhibit L5 PCs via ascending axons. **(B)** Excitatory translaminar pathways recruit L5 interneurons. Excitatory cells in L2/3, L4, and L6 can target PV/basket cells. Additionally, L2/3 PCs synapse onto L5 SOM/Martinotti cells.

In addition to intralaminar circuits, L5 is subject to a number of inhibitory influences from other cortical layers in the local circuit. Translaminar inhibition of L5 can derive either from input from GABAergic cells in other cortical layers that target L5 PCs, or from the synaptic activation of GABAergic cells within L5 through translaminar excitatory afferents. With respect to the former scenario, studies using axonal reconstruction, paired intracellular recordings, and viral tracing methods indicate that L5 PCs receive input from a substantial number of interneurons in L2/3, L4, and L6, albeit at a lower frequency than from L5 interneurons (Helmstaedter et al., 2009a,b,c; Jiang et al., 2013; Lee et al., 2015; DeNardo et al., 2015; Figure 2A). Connections from several different varieties of interneurons in layers 1 and 2/3 have been observed onto L5 PCs (Jiang et al., 2013, 2015). Interestingly, different L2/3 interneurons appear to target specific dendritic domains of L5 PCs (Jiang et al., 2013), a result which has been mirrored by recent work in the hippocampus (Bloss et al., 2016). Since dendritic inhibition can be quite spatially precise (Chiu et al., 2013; Marlin and Carter, 2014; Müllner et al., 2015), this organization might allow for cell-type specific control over the distinct spatial domains of the various types of dendritic spiking that occur in L5 PCs (Larkum et al., 2009). Studies in mouse V1 have described a translaminar inhibitory circuit in which a subclass of L6 PV neurons can powerfully suppress PCs across all other cortical layers, including L5 (Olsen et al., 2012; Bortone et al., 2014). If this circuit also exists in the barrel cortex, it could play a major role in L5 gain control during sensory activity.

Despite the existence of these direct translaminar inhibitory pathways, optogenetic experiments mapping inhibitory inputs to L5 PCs (either from GABAergic non-specifically, or selectively from PV neurons) suggest that inhibition onto L5 PCs most strongly originates from interneurons within L5 (Schubert et al., 2001, 2006; Brill and Huguenard, 2010; Kätzel et al., 2011; Pluta et al., 2015). Thus the major source of translaminar inhibition to L5 PCs is more likely to be through the synaptic recruitment of L5 interneurons by afferent axons from other cortical layers (Figure 2B).

With the exception of L1, all cortical layers make excitatory axonal projections onto L5, which can recruit L5 interneurons to fire. This recruitment, often termed “feed-forward inhibition” (FFI), is a nearly ubiquitous motif in cortical microcircuits where afferent excitatory input simultaneously recruits local inhibitory neurons that provide inhibition to the same cells receiving excitation. Circuits for FFI have been well studied at the projections from thalamus to L4 (Gibson et al., 1999; Bruno and Simons, 2002; Swadlow, 2003; Gabernet et al., 2005; Wilent and Contreras, 2005; Sun et al., 2006; Cruikshank et al., 2007, 2010; Daw et al., 2007), and from L4 to L2/3 (Helmstaedter et al., 2008; Xu and Callaway, 2009; House et al., 2011; Adesnik et al., 2012; Elstrott et al., 2014; Li et al., 2014; Xue et al., 2014). In these pathways, incoming axons primarily recruit PV interneurons in the recipient layer. PV-mediated FFI serves many functions: it enhances the temporal specificity of incoming signals (Pouille and Scanziani, 2001; Gabernet et al., 2005), extends the dynamic range of the downstream population response (Pouille et al., 2009), and imposes a synchrony filter on incoming inputs (Bruno, 2011). FFI is surprisingly poorly understood in L5. We recently described a translaminar inhibitory circuit in which descending excitatory axons from L4 synapses on L5 PV neurons, which in turn drives inhibition in L5 PCs (Pluta et al., 2015). FFI mediated through this circuit has the net effect of suppressing L5 PCs, sharpening their spatial tuning to tactile stimuli. Although it remains unexplored, we hypothesize that the L4-L5 translaminar inhibitory circuit may also be crucial for the precise timing of touch-evoked spiking in L5 PCs. Another recent study found that L6 PCs can drive disynaptic inhibition onto L5 PCs, specifically to PCs in L5a (likely IT-type PCs, Kim et al., 2014). Interestingly, both of these studies found that afferent input to L5 was much more effective at driving PV neurons than Martinotti cells. Thus inhibition in the L4→L5 and L6→L5 pathways appears to be organized similarly to other translaminar circuits by operating through PV neurons.

Notably, there is a gap in our understanding of FFI in the L2/3 to L5 pathway, one of the densest projections in the cortical microcircuit. Stimulation of L2/3 generates robust FFI in L5 PCs (Pouille et al., 2009; Adesnik and Scanziani, 2010), but preliminary evidence suggests that the mechanisms of L2/3→L5 FFI may be unique. Surveys of connectivity onto L5 PV cells in S1 (Jin et al., 2014; Pluta et al., 2015), V1 (Jiang et al., 2015), and other cortical areas (Otsuka and Kawaguchi, 2009; Apicella et al., 2012) expose only moderate excitatory input from L2/3. In contrast, L5 Martinotti cells appear to receive strong input from L2/3 (Kapfer et al., 2007; Otsuka and Kawaguchi, 2009; Apicella et al., 2012; Jiang et al., 2015). This suggests that L2/3→L5 FFI may operate primarily through SOM, rather than PV neurons, and thus predominantly routes inhibition to the L5 PC dendrites, similar to what occurs between the olfactory bulb and the piriform cortex (Stokes and Isaacson, 2010). Because of the filtering cable properties of the apical dendrites, dendritic FFI is likely suboptimal for enforcing rigid temporal control over the somatic spiking of L5 PCs *per se*, but is instead poised to directly regulate the integration of excitatory inputs from L2/3 PCs to the apical compartment of an L5 PC.

## Interareal Recruitment of L5 Inhibition

Long-range axons from the thalamus, motor cortex, and contralateral S1 that innervate L5 PCs, also recruit L5 inhibitory neurons. For example, ascending axons from primary thalamus ventral posteromedial nucleus, (VPM) can recruit both L5 PV and SOM cells driving various forms of FFI. Interestingly, as described for FFI circuits in hippocampal CA1 (Pouille and Scanziani, 2004), during sustained activity, VPM afferents will initially recruit L5 PV cells, but then switch to driving a subpopulation of L5 non-Martinotti SOM cells labeled by the X94 line (Tan et al., 2008), although it is still unclear if these L5 ×94 neurons project inhibition onto L5 PCs. In contrast, L5 Martinotti cells are not driven by VPM afferents and thus do not appear to participate in thalamocortical FFI (Cruikshank et al., 2010; Ji et al., 2015). Motor cortex afferents to S1 appear to target L5 PV neurons and not L5 Martinotti cells (Kinnischtzke et al., 2014), whereas inputs from contralateral cortex drive inhibition in L5 PCs through at least two distinct circuits. First, callosal projections likely drive FFI using the familiar motif of activating L5 PV neurons, though this has not yet been directly demonstrated in barrel cortex (Karayannis et al., 2006; Lee et al., 2014; Rock and Apicella, 2015). However, these projections also drive an unusual form of slow, GABA_B_-dependent inhibition onto the dendrites of L5 PCs, which is mediated by the recruitment of neurogliaform cells in layer 1 (Palmer et al., 2012, 2013). Because callosal projections can synapse onto both the basal and apical compartments of L5 PCs, these dual pathways may allow FFI to be spatially matched to the same subcellular compartments as feed-forward excitation. As many other long-range inputs also form synapses at the distal apical tufts of L5 PCs (Cauller and Connors, 1994; Cauller et al., 1998; Larkum and Zhu, 2002; Petreanu et al., 2009, 2012; Mao et al., 2011; Xu et al., 2012; Manita et al., 2015), it will be interesting to see if these circuits also possess similar capabilities to generate dendritic inhibition.

How might inhibition contribute to communication in these long-range circuits? Long-range afferents to S1 can convey a variety of information, including data on whisker kinematics (Curtis and Kleinfeld, 2009; Petreanu et al., 2012), attentional focus (Harris, 2013; Zagha et al., 2013), and cognitive features in decision-based tasks (Yang et al., 2016). FFI in these circuits will powerfully influence how activity in these afferent pathways is integrated with ongoing local cortical activity. Do these circuits incorporate all L5 PCs in the same manner? PT and IT cells both send and receive long-range inputs in a cell-type specific manner. For example, since the axons of PT cells do not cross the corpus callosum, only L5 IT cells will be able to directly activate the circuits for interhemispheric inhibition mentioned above. Furthermore, while both PT and IT cells receive long-range callosal input, two recent studies found that optogenetic activation of callosal fibers generates inhibition in a manner specific to cell-type. Interestingly, this effect appears to be regionally specialized. In auditory cortex IT neurons are more strongly inhibited, while in prefrontal cortex PT neurons are more strongly inhibited (Lee et al., 2014; Rock and Apicella, 2015). This type of specialization is intriguing, since it might provide a means to direct and gate the flow of activity between different regions of the cortex. More research is needed to determine if similar specificity is seen in other cortical areas or other types of long-range circuits.

## Future Directions

Many recent advances in the understanding of cortical inhibitory circuits can be attributed to the widespread adoption of cell-type specific Cre recombinase driver lines (Taniguchi et al., 2011). These lines have allowed investigators to target subsets of cortical interneurons with optical reporters for targeted electrophysiological recording or functional imaging. Experiments using these lines *in vivo* have revealed how the various interneuron subtypes modulate their responses to different sensory stimuli (Kerlin et al., 2010; Ma et al., 2010; Runyan et al., 2010; Bock et al., 2011; Hofer et al., 2011; Adesnik et al., 2012; Gentet et al., 2012; Scholl et al., 2015) and shifting behavioral states (Lee et al., 2013; Pi et al., 2013; Polack et al., 2013; Fu et al., 2014; Reimer et al., 2014). By expressing optogenetic actuators rather than reporters in these inhibitory cell types, many groups have begun to manipulate interneurons with cell-type specificity to assess their specific contributions to cortical computation and behavior (Adesnik et al., 2012; Atallah et al., 2012; Lee et al., 2012; Wilson et al., 2012; Seybold et al., 2015).

Nevertheless, the recent and widespread reliance on just a few Cre lines (particularly the PV- SOM- and VIP-Cre lines) can also lead to unintended problems. First, these lines are not completely non-overlapping. Up to ~10% of Cre expressing cells in the widely used SOM-IRES-Cre line may actually be PV expressing, fast-spiking interneurons (Hu et al., 2013). Second, the availability of only a few Cre driver lines for interneurons, along with the paucity of more specific alternatives, has ushered in a new era of “lumping” in the study of interneurons. While this reunification is conceptually beneficial in many ways, it is undoubtedly an over simplification; each of these major subtypes (PV, SOM and 5HT3aR) are highly heterogeneous groups, as described above; for example, the PV-Cre line labels both basket and chandelier cells, while the SOM-Cre line labels both Martinotti and X94 neurons. Third, and perhaps most problematically, all of these Cre lines label interneurons non-specifically across all cortical layers. This is particularly an issue for *in vivo* optogenetic manipulations where one-photon illumination will non-specifically activate or suppress Cre-expressing interneurons across all cortical layers. This makes it difficult to ascribe results gained with these manipulations to interneurons in specific lamina.

However, there are potential solutions to these problems. The first would be the development and adoption of new transgenic lines that target highly specific inhibitory cell types (Taniguchi et al., 2011; Shima et al., 2016). In the short term, further characterizing existing Cre-driver lines that might fractionate the three major interneuron subgroups, such as the Chrna2-Cre line for SOM cells (Leão et al., 2012), or retrofitting existing highly specific GFP lines for Cre expression (Tang et al., 2015), could be extremely valuable. In the long term, advances in single cell transcriptional profiling of cortical neurons could provide quantitative genetic data which may aid in the much more complete delineation of new GABAergic cell types (Armañanzas and Ascoli, 2015; Zeisel et al., 2015; Tasic et al., 2016). At the same time, these data will hopefully identify highly differentially expressed genes between the various subtypes that will provide genetic handles to develop transgenic lines (including Cre reporters) that will permit targeting and optogenetic control of many more subclasses of interneurons. While doing so may depend on sophisticated intersectional strategies involving multiple recombinases, such technology already exists (Fenno et al., 2014; Madisen et al., 2015; reviewed in Huang, 2014).

As we alluded to above, the recent explosion of functional information on inhibitory subtypes *in vivo* has mostly focused on superficial interneurons due to the technical difficulty of imaging deeper cells in the cortex due to light scattering, and the practical challenges of maneuvering recording pipettes into deeper layers under visual guidance. A number of recent technical advantages should overcome these challenges. These include adaptive optics for deeper *in vivo* imaging (Wang et al., 2015), implantable optics such as prisms (Andermann et al., 2013) or GRIN lenses (Barretto et al., 2009), three photon imaging (Horton et al., 2013), regenerative amplification of ultrafast pulses (Mittmann et al., 2011), and red-shifted fluorophores that are much less affected by optical scattering in brain tissue. Recent advances in red-shifted calcium and voltage dyes hold particular promise (Inoue et al., 2014; Gong et al., 2015; Huang et al., 2015).

New optical methods will also open new avenues for manipulation of interneuron activity. Novel techniques for spatial light modulation are poised to provide unprecedented optogenetic precision, enabling experimenters to arbitrarily target manipulations to single cells or even specific ensembles of neurons (Bovetti and Fellin, 2015). Furthermore, these manipulations are flexible and can be altered online, opening the door to new methods for adaptive/closed-loop experiments to probe network dynamics and structure (Grosenick et al., 2015). However, even in the best of circumstances it is not straightforward to interpret optogenetic perturbations of neurons embedded in recurrent networks (Kumar et al., 2013; Seybold et al., 2015). To account for this, it will be necessary to embrace more complicated, nonlinear models of cortical dynamics and to incorporate these into experimental design (Rubin et al., 2015; Litwin-Kumar et al., 2016). By combining these new technologies with much more specific and sophisticated genetic tools, it should be possible to make considerable progress in understanding the function and impact of L5 interneurons in the coming few years.

## Author Contributions

AN and HA researched, wrote, and edited the manuscript.

## Conflict of Interest Statement

The authors declare that the research was conducted in the absence of any commercial or financial relationships that could be construed as a potential conflict of interest.
