# Supplementary material for: Inhibitory Circuits in Cortical Layer 5
Source: Front Neural Circuits. 2016 May 6;10:35. doi: 10.3389/fncir.2016.00035 (PMC4859073; doi:10.3389/fncir.2016.00035)
Supplement: Supplementary file 1 [file Table_1.docx]

Supplementary Table 1. Properties and connectivity of layer 5 GABAergic interneurons. Boxes containing a question mark indicate areas where direct experimental evidence is not available or lacking

| **Type**  (other common nomenclatures) | **Parvalbumin (PV) expressing interneurons**  (Fast-spiking, narrow-spiking, thin-spiking) | | | **Somatostatin (SOM) expressing interneurons**  (Low-threshold spiking, regular spiking nonpyramidal) | | | **Serotonin receptor (5HT3aR) expressing interneurons**  (Irregular spiking) | | |
| --- | --- | --- | --- | --- | --- | --- | --- | --- | --- |
| **Subtype** | **Basket** | **Chandelier** | | **Martinotti** | **X94** | | **VIP** | **Neurogliaform cells** | |
| **Fraction of L5 interneurons**  (Gonchar, 2008; Lee et al., 2010; Meyer et al., 2011; Rudy et al., 2011; Sohn et al., 2014; Xu et al., 2010) | 45-60% | | | 20-50% | | | 5-15% | | |
| **Morphology**  (Buchanan et al., 2012; Ma et al., 2006; Oláh et al., 2009; Prönneke et al., 2015; Silberberg and Markram, 2007; Taniguchi et al., 2013; Wang et al., 2002, 2004; Xiang, 1998) | Bi/multipolar dendritic morphology  Dense axonal arbor, can be intra or translaminar. Axons target the perisomatic compartment of PCs | | Bi/multipolar dendritic morphology  Dense, hanging axonal arbor. Cartridge-like synapses onto axon initial segments give the appearance of a chandelier | Bitufted or bi/multipolar dendrites  Radially oriented, translaminar axon ascending to layer 1. Innervates distal dendrites. | | Bitufted or bi/multipolar dendrites  Radially oriented, translaminar axon densely targets a single barrel in L4 but avoids L1 | Radially oriented, bipolar/bitufted dendrites  Radially oriented axons, mostly restricted to infragranular layers. Target dendrites of PCs but somata of PV neurons | | Multipolar dendrites  Dense, local axon |
| **Molecular Markers**  (Gonchar, 2008; Huang, 2014; Kubota et al., 2011; Leão et al., 2012; Ma et al., 2006; Rudy et al., 2011; Sohn et al., 2014; Xu et al., 2010) | PV | | | SOM  Preprodynorphin  Calretinin  NPY | | | 5HT3aR  NPY  CCK  Calretinin | | |
|  |  |  |  | Chrna2  Calbindin | |  | VIP | | Reelin  nNOS |
| **Intrinsic properties**  (Galarreta and Hestrin, 2002; Ma et al., 2006, 2012; Markram et al., 2004; Oláh et al., 2009; Prönneke et al., 2015) | Fast-spiking  Non-adapting  Large, fast AHP  Low input resistance | | | Regular, adapting spiking,  High input resistance,  Can perform a low-threshold spike on rebound from hyperpolarization | | Quasi fast-spiking phenotype, Some adaptation  Low input resistance,  No low-threshold spike | Regular or irregular spiking,  Adapting  High input resistance | | Late spiking or irregular spiking  Adapting |
| **Local excitatory inputs**  (Angulo et al., 2003; Apicella et al., 2012; Le Bé et al., 2007; Buchanan et al., 2012; Jiang et al., 2015; Jin et al., 2014; Kapfer et al., 2007; Kim et al., 2014; Marques-Smith et al., 2016; Otsuka and Kawaguchi, 2009; Pluta et al., 2015; Silberberg and Markram, 2007; Staiger et al., 2002) | L2/3, L4, L5 (PT and IT), L6 CT | | ? | L5 PT  L2/3 | | ? | L5 PT | | ? |
| **Long-range inputs**  (Cruikshank et al., 2010; Ji et al., 2015; Karayannis et al., 2006; Kinnischtzke et al., 2014; Lee et al., 2013; Rock and Apicella, 2015; Sun et al., 2013; Tan et al., 2008; Zhang et al., 2014) | VPM, M1, contralateral S1 | | | ? | | VPM | Long-range corticocortical inputs in L2/3, unknown in L5 | | ? |
| **Synaptic targets**  (Blazquez-Llorca et al., 2014; Buchanan et al., 2012; Hioki et al., 2013; Jiang et al., 2015; Kruglikov and Rudy, 2008; Ma et al., 2006; Packer and Yuste, 2011; Pfeffer et al., 2013; Silberberg and Markram, 2007; Walker, 2016; Xiang et al., 2002; Xu et al., 2013) | PCs, other PV cells, Martinotti  Mostly but not exclusively intralaminar | | PCs, other PV cells  Mostly intralaminar | All neurons except other SOM cells  Intralaminar or translaminar | | ?  Neurons in L4  Preferentially target PV cells | PV, SOM | | Chemical and electrical connectivity with all interneurons, intralaminar |
